# Emotionally intelligent people reappraise rather than suppress their emotions

**DOI:** 10.1371/journal.pone.0220688

**Published:** 2019-08-12

**Authors:** Alberto Megías-Robles, María José Gutiérrez-Cobo, Raquel Gómez-Leal, Rosario Cabello, James J. Gross, Pablo Fernández-Berrocal

**Affiliations:** 1 Department of Basic Psychology, Faculty of Psychology, University of Málaga, Málaga, Spain; 2 Department of Developmental and Educational Psychology, Faculty of Psychology, University of Granada, Granada, Spain; 3 Department of Psychology, Stanford University, Stanford, CA, United States of America; Technion Israel Institute of Technology, ISRAEL

## Abstract

It has long been thought that emotional intelligence (EI) involves skillful emotion regulation, but surprisingly little is known about the precise links between EI and emotion regulation. To address this gap in the literature, we examined the relation between EI—operationalised as an ability—and the use of two common emotion regulation strategies—cognitive reappraisal and expressive suppression. Seven hundred and twelve participants from a community sample in Spain were assessed on ability EI (using the MSCEIT) and emotion regulation (using the ERQ). Findings revealed that EI ability was positively associated with cognitive reappraisal and negatively associated with expressive suppression. These relationships were moderated by gender and age. The strength of the association between EI and cognitive reappraisal increased with age for men, while this strength decreased with age for women. Conversely, the strength of the association between EI and suppression decreased with age for men, but increased with age for women. These findings confirm the expectation that EI is associated with greater use of generally adaptive forms of emotion regulation (reappraisal), and lesser use of generally maladaptive forms of emotion regulation (suppression), although effect sizes were quite modest. Observed differences in the strength of associations between EI and emotion regulation may be the result of gender differences in the development of emotional skills along with cultural changes in emotional education and social norms.

## Introduction

Skillful emotion regulation is essential for healthy adaptation, and has been associated with positive outcomes in the domains of affect, social functioning, and well-being [1, 2]. Two of the most commonly used and widely studied emotion regulation strategies are cognitive reappraisal and expressive suppression [3, 4].

Cognitive reappraisal is defined as a form of cognitive change that involves a reinterpretation of an emotion-eliciting situation in order to modify its emotional impact [5]. For example, in a potentially stressful situation, such as an argument with a friend, we might reevaluate the situation from the point of view of the other person, thereby decreasing the emotional burden. On the other hand, expressive suppression is a form of response modulation that entails the inhibition of an ongoing emotion-expressive behavior while the individual is already emotionally aroused [5]. An example of this would be suppressing our negative emotional responses when our boss criticizes our work.

Cognitive reappraisal is regarded as a generally adaptive strategy, whereas expressive suppression is regarded as a generally less adaptive strategy. This idea is consistent with findings showing that, compared with people who tend to suppress their emotions, people who use reappraisal strategies more frequently experience more positive and less negative emotions, are more successful at mood repair, have better social relationships, better psychological health, suffer depressive symptoms less frequently, and show greater self-esteem and life satisfaction [1, 6, 7, 8].

One construct that is often linked to the emotional regulation style adopted by each individual is emotional intelligence (EI). EI is defined by Mayer & Salovey as “*the ability to perceive accurately*, *appraise*, *and express emotion; the ability to access and/or generate feelings when they facilitate thought; the ability to understand emotion and emotional knowledge; and the ability to regulate emotions to promote emotional and intellectual growth*” [9]. This definition suggests that one of the key aspects of EI is the ability to successfully regulate emotions, but it does not specify what emotion regulation processes are (and are not) used by high-EI individuals.

This expectation has been born out in several studies focusing on cognitive reappraisal and expressive suppression [10]. Cabello et al. [7], with a Spanish community sample of 423 participants and using the TMMS to assess EI, a self-report trait EI scale, observed that a higher level of EI was associated with a more frequent use of reappraisal and less frequent use of suppression. Schutte, Manes, & Malouff [11] found similar results using the Assessing Emotions Scale (a self-report trait EI measure) with an Australian community sample of 73 participants and by Śmieja & Kobylińska [12] using the TIE (performance-based ability EI measure) with a sample of 349 Polish undergraduate students. In a cross-cultural study using 502 European-American participants and 538 Japanese participants, Nozaki [13] observed that reappraisal was positively related to EI level in both cultures, however, suppression was negatively related to EI level in European-American, but not in Japanese. The EI assessment instrument used by Nozaki [13] was the TEIQue-SF, a self-report trait EI scale. Andrei, Smith, Surcinelli, Baldaro, & Saklofske [14], in a psychometric study exploring the criterion validity of the TEIQue, a self-report trait EI questionnaire, showed with a sample of 227 participants that higher scores in EI were related to lower suppression, but there was no evidence of relationship with reappraisal. Finally, Kafetsios & Loumakou [15], using the the EQ-I, a self-report trait EI measure, with a sample of 485 Greek teachers, found no clear evidence of a connection between EI and the emotion regulation strategies. Of the four key EQ-i dimensions, only the intrapersonal EI was negatively correlated with suppression and the interpersonal EI positively with reappraisal.

One limitation of these studies is that in most cases they have been based on self-report measures or have treated EI as a broad personality trait as opposed to an ability. Śmieja & Kobylińska [12] was the only study to assess EI by a performance-based ability measure, but the questionnaire used (TIE) is unusual in the literature and is in the Polish language [16]. Understanding EI as an ability rather than as a personality trait would seem particularly useful when studying abilities such as the type of emotion regulation strategies used. Moreover, the previous literature has revealed a lack of a relationship between self-reports and performance measures of EI [17].

With respect to the influence of individual variables such as gender and age on the relationship between EI and emotion regulation strategies, Śmieja & Kobylińska [12] observed that in men, EI abilities were related to the use of suppression, but this was not the case in women. The influence of age on this relationship remains unknown in the literature. In addition, it has been shown that, individually, both EI and emotion regulation are sensitive to changes across the lifespan and gender. Men show lower EI and a higher use of suppression strategies than women, but no gender differences are found in the use of reappraisal strategies [7, 18]. With respect to age, there is no clear consensus; while some studies have found that older people have better EI abilities than young people [19, 20], other studies have shown no relationship [21]. A similar situation is observed with emotion regulation: some studies have shown that as people get older they increase the use of reappraisal and decrease the use of suppression strategies [1], but there are also studies showing an age-related maintenance [22].

The aim of the present study was to clarify the relationship between EI—operationalized as an ability—and the use the cognitive reappraisal and expressive suppression. Unlike previous studies, we employed the most popular and well-established performance measure for assessing ability EI: The Mayer-Salovey-Caruso emotional intelligence test (MSCEIT) [23]. The study was carried out in a community sample with individuals of different ages, which allows us to generalize our findings to a wider population in comparison with previous studies. Finally, given the influence of age and gender on the link between emotion regulation and EI abilities, we also decided to explore the moderating effect of these variables.

## Method

### Participants

Seven hundred and twelve volunteer participants took part in this experiment (442 women and 270 men). They were recruited through advertisements in higher education centers across Spain. The average age was 36.56 years (SD = 15.11), ranging between 18 and 76. All participants signed a written informed consent form and they were treated in accordance with the Helsinki declaration. All data collected were confidential and anonymous. The Research Ethics Committee of the University of Málaga approved the study protocol as part of the project PSI2017-84170 (IRB approval number 10-2018-H).

### Measures

The MSCEIT is a performance-based ability measure of EI [23]. The MSCEIT is composed of 141 items divided into four branches according to Mayer and Salovey’s theory: perceiving, facilitating, understanding, and managing emotions [9]. The instrument provides separate scores for each branch and an overall score. EI abilities were measured using the Spanish version of the MSCEIT [24], which has shown good internal consistency (α = .95).

The Emotion Regulation Questionnaire (ERQ) [3] is a self-report questionnaire assessing two emotion regulation strategies: cognitive reappraisal and expressive suppression. This instrument is composed of 10 items, in which participants must indicate their degree of agreement with the statement on a 7-point Likert scale. Four items are related to suppression and 6 items to reappraisal. We used the Spanish version of the ERQ [7], which has an adequate internal consistency similar to the English version (α = .75 for expressive suppression, α = .79 for cognitive reappraisal).

### Statistical analysis

First, Pearson’s correlations were calculated to describe the relationships between MSCEIT and ERQ subscales. Second, t-tests for gender and correlation analyses for age were conducted to explore the influence of these factors on MSCEIT and ERQ. Third, a moderation model was employed to study the conditional effect of gender and age on the relationship between MSCEIT and ERQ. Moderating effects were tested by SPSS PROCESS 3.1 (Model 3, mean-centered variables) [25].

## Results

Pearson's correlation analyses revealed a negative relationship between MSCEIT and expressive suppression (r = -.25, *p* < .001) and a positive relationship between MSCEIT and cognitive reappraisal (r = .16, *p* < .001). Individuals with higher EI levels use fewer suppression strategies and more reappraisal strategies. Correlations for MSCEIT branches are available as Supplementary Material (S1 Table). It is noteworthy that the MSCEIT branch showing the strongest correlation with the ER strategies was the emotion management branch.

Focusing on the effect of gender and age, compared with men, women showed higher scores on MSCEIT (*p* < .001) and cognitive reappraisal (*p* = .04), and lower scores on expressive suppression (*p* < .001). As age increased there was a decrease in the MSCEIT scores (*p* < .001) and an increase in expressive suppression (*p* < .001; see S2 Table) for more details on gender and age results.

**Fig 1:**
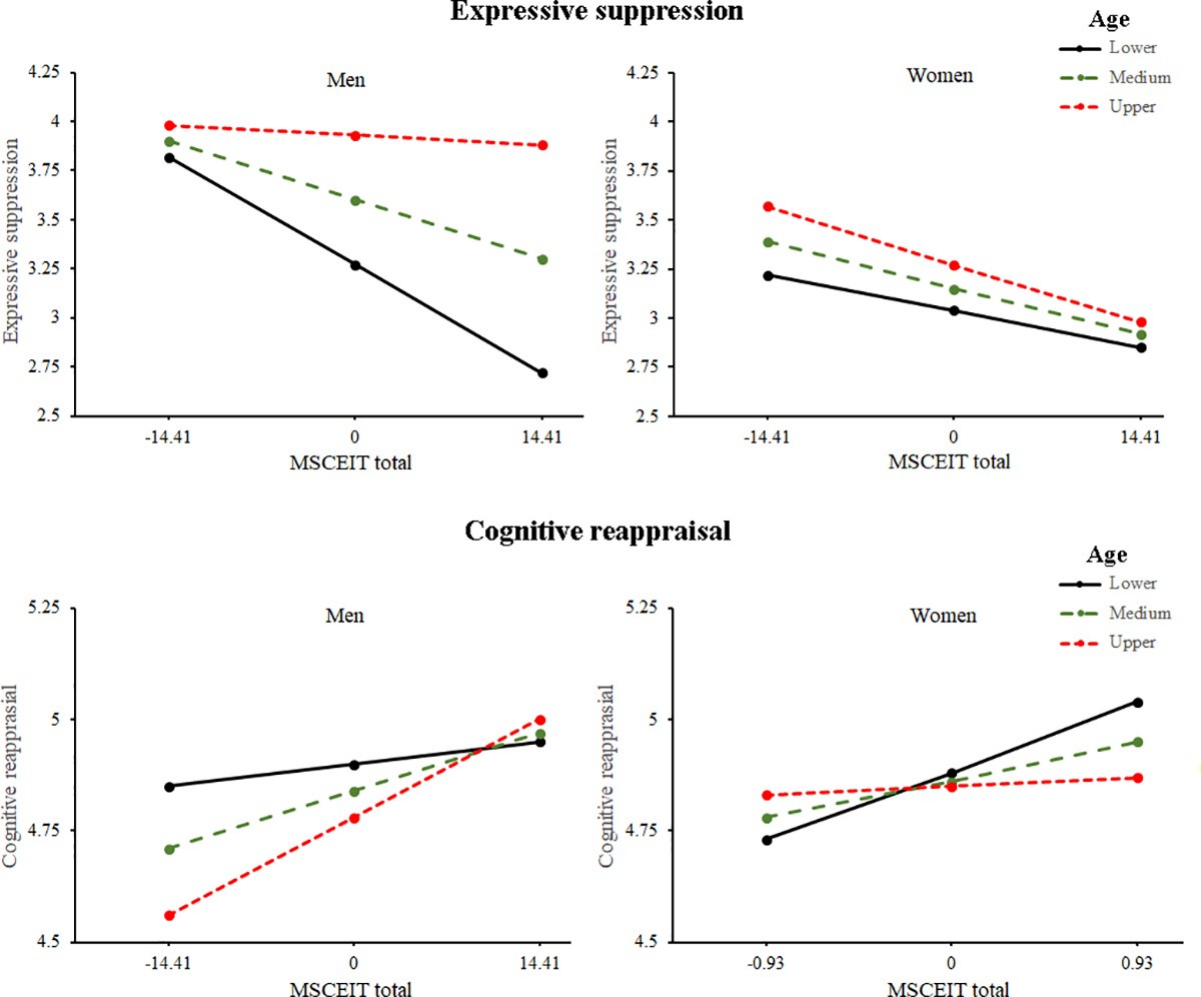
Top panel: moderation of gender and age on the relationship between MSCEIT total and expressive suppression (top panel) and on the relationship between MSCEIT total and cognitive reappraisal (bottom panel). Values of age, expressive suppression, and cognitive reappraisal are mean-centered values.

With respect to the moderation analyses with expressive suppression as dependent variable, these revealed a three-way interaction between MSCEIT, age, and gender (interaction coefficient = -.0014, 95% CI [-.00226 -.00055]). To further study this moderation, we estimated the conditional effect of these variables using the pick-a-point approach at three age values for each gender [26]: lower age (mean - 1SD), medium age (mean), and upper age (mean + 1SD). The results revealed that the negative relationship between MSCEIT and expressive suppression decreased with age, disappearing in the upper age group. Women showed this relationship in the three age groups, and unlike men, the relationship was stronger as age increased, although these changes were small (Table 1 and Fig 1).

**Table 1:** Conditional effect of age and gender on the relationship between total MSCEIT and expressive suppression, and between MSCEIT understanding and cognitive reappraisal.

		MSCEIT total Expressive suppression		MSCEIT total Cognitive reappraisal	
Gender	Age	Effect	95% CI [lower, upper]	Effect	95% CI [lower, upper]
Men	Lower	-.0381	[-.0560, -.0203]	.0034	[-.0106, .0175]
Men	Medium	-.0206	[-.0317, -.0096]	.0093	[.0006, .0180]
Men	Higher	-.0031	[-.0146, .0083]	.0152	[.0062, .0242]
Women	Lower	-.0127	[-.0233, -.0020]	.0109	[.0026, .0193]
Women	Medium	-.0165	[-.0249, -.0080]	.0061	[-.0005, .0128]
Women	Higher	-.0203	[-.0329, -.0077]	.0014	[-.0085, .0113]

On the other hand, the moderation analysis for cognitive reappraisal also revealed a three-way interaction between MSCEIT, age, and gender (interaction coefficient = -.0007, 95% CI [-.00137 -.00003]). The pick-a-point procedure showed that for men the positive relationship between MSCEIT and cognitive reappraisal was stronger as age increased, while for women this relationship weakened with age (Table 1 and Fig 1). Additional moderated moderation analyses for MSCEIT branches are presented in S3 Table.

## Discussion

Previous studies have shown that greater use of cognitive reappraisal and lesser use of expressive suppression is associated with more positive consequences in the domains of affect, social functioning, and well-being [1, 2, 7]. The present study supports the notion that the employment of these emotion regulation strategies is related to EI abilities.

Our findings revealed that higher EI abilities were associated with a greater use of cognitive reappraisal strategies and a lesser use of expressive suppression strategies for regulating emotions. These results are in accord with the findings of other studies in the literature using self-reports and less established measuring instruments than the MSCEIT [7, 11, 12]. Reappraisal strategies involve modulating the emotion-generative process before emotional response tendencies have been generated [1, 5]. As Śmieja & Kobylińska [12] propose, modification of the emotional sequence in such early phases requires the adequate perception, facilitation, understanding, and regulation of emotion abilities (EI abilities). In this regard, high EI individuals should be able to modulate their emotions from the beginning of the generative process, but low EI individuals would be forced to suppress them once the emotion had already arisen, given their lower capacity to regulate them earlier. Of course, there are situations where suppressing is the best or the only option of emotional regulation (e.g. when there is not enough time to reappraise). Nonetheless, problems arise with the excessive use of suppression strategies in our daily life given that this process modifies emotion-expressive behavior without reducing the emotional experience itself. This, moreover, requires an additional cognitive effort to maintain the behavioral aspects of the emotional response that is continuously suppressed [1]. Thus, individuals with higher EI would prefer to employ more adaptive reappraisal strategies rather than simply attempting to suppress their emotional responses.

In addition, we observed a moderator effect of age and gender on the association between EI abilities and emotion regulation strategies. The negative relationship between EI and suppression strategies decreased with age for men, but slightly increased for women. Conversely, the positive relationship between EI and cognitive reappraisal increased with age for men but decreased with age for women. These differences in the pattern of results could, in part, be explained by the differential development of the emotion regulation strategies between genders. Previous studies have shown that women possess higher emotional abilities and use a greater range of regulation strategies when compared with men [27]. Thus, women may make less use of suppression strategies since youth. Moreover, following Śmieja & Kobylińska [12], the fact that the relationship between EI and reappraisal disappears as women get older could be because they learn and begin to use other regulation strategies (which occurs to a lesser extent in men). On the other hand, a further possible explanation—but not exclusive of the previous proposal—is based on generational factors. Emotional education has become an issue of relevance in recent decades and changes in this direction have been introduced into the education system to improve the emotional skills of the population. These educational changes could have modified the traditional gender roles and social norms associated with emotional regulation behavior [28]. This idea could explain why in our sample the younger men were those who best regulated their emotions, obtaining, in the case of those with high EI, scores close to the women.

One important limitation of the present study is that our findings are based on correlational analyses in a cross-sectional design. In order to establish causal relationships between the studied variables, further research should include longitudinal experimental studies. Moreover, in future studies it would be interesting to evaluate a wider range of regulation strategies.

In conclusion, this study shows that individuals with different EI levels differ in the use of emotion regulation strategies. Higher EI people showed a greater use of cognitive reappraisal, a strategy that has been shown to be more adaptive for regulating emotions when compared with expressive suppression. In contrast, it was found that people with lower EI employ the latter strategy more frequently. Our results also revealed that this relationship appears to depend on both age and gender. These factors need to be considered in future studies of emotion regulation processes and their implementation in intervention programs for promoting better psychological health, social functioning, and life satisfaction.

## Supporting information

S1 File Dataset. (SAV)

S1 Table Pearson correlations between the MSCEIT branches and the emotion regulation strategies from ERQ. (DOCX)

S2 Table Descriptive statistics (mean and standard deviation), T-tests comparing genders (T value and Cohen’s d), and Pearson’s correlations with age for the MSCEIT total, MSCEIT branches and ERQ variables. (DOCX)

S3 Table Conditional effect of age and gender on the relationship of the perceiving and facilitating MSCEIT branches with expressive suppression, and on the relationship of MSCEIT understanding with cognitive reappraisal. Rest of moderated moderation analyses showed no significant three-way interaction.
(DOCX)
