# Supplementary material for: Emotionally intelligent people reappraise rather than suppress their emotions
Source: PLoS One. 2019 Aug 12;14(8):e0220688. doi: 10.1371/journal.pone.0220688 (PMC6690525; doi:10.1371/journal.pone.0220688)
Supplement: S1 Table — (DOCX) [file pone.0220688.s002.docx]

S1 Table. Pearson correlations between the MSCEIT branches and the emotion regulation strategies from ERQ.

|  | MSCEIT facilitating | MSCEIT understanding | | MSCEIT managing | Cognitive reappraisal | Expressive suppression | |
| --- | --- | --- | --- | --- | --- | --- | --- |
| MSCEIT perceiving | .50** | .26** | | .36** | .05 | -.10** | |
| MSCEIT facilitating |  | .26** | | .36** | .09* | -.20** | |
| MSCEIT understanding |  |  | | .42** | .16** | -.23** | |
| MSCEIT managing |  |  | |  | .19** | -.24** | |
| Cognitive reappraisal |  |  | |  |  | .04 | |
| *Note: p* < .05*, *p* < .01** | | |  | | | |  |
