# Supplementary material for: Emotionally intelligent people reappraise rather than suppress their emotions
Source: PLoS One. 2019 Aug 12;14(8):e0220688. doi: 10.1371/journal.pone.0220688 (PMC6690525; doi:10.1371/journal.pone.0220688)
Supplement: S2 Table — (DOCX) [file pone.0220688.s003.docx]

S2 Table. Descriptive statistics (mean and standard deviation), T-tests comparing genders (T value and Cohen’s d), and Pearson’s correlations with age for the MSCEIT total, MSCEIT branches and ERQ variables.

|  | Mean (SD) for men | Mean (SD) for women | T-test between genders (Cohen’s d) | Correlation with age in men (Pearson’s r) | Correlation with age in women (Pearson’s r) |
| --- | --- | --- | --- | --- | --- |
| MSCEIT total | 96.47 (14.11) | 102.15 (14.12) | 5.20** (0.40) | -.18** | -.39** |
| Cognitive reappraisal | 4.76 (0.95) | 4.91 (0.91) | 1.98* (0.22) | -.11 | -.05 |
| Expressive suppression | 3.71 (1.19) | 3.12 (1.22) | 6.36** (0.50) | .26** | .17** |
| MSCEIT perceiving | 98.80 (14.80) | 100.73 (14.07) | 1.74 (0.13) | -.07 | -.16** |
| MSCEIT facilitating | 97.14 (14.04) | 101.74 (14.31) | 4.18** (0.32) | -.16** | -.30** |
| MSCEIT understanding | 97.33 (14.68) | 101.63 (13.95) | 3.91** (0.30) | -.29** | -.44** |
| MSCEIT managing | 97.74 (15.08) | 103.63 (13.62) | 5.37** (0.43) | -.02 | -.31** |
| *Note: p* < .05*, *p* < .01** | | | | | |
