# Supplementary material for: Emotionally intelligent people reappraise rather than suppress their emotions
Source: PLoS One. 2019 Aug 12;14(8):e0220688. doi: 10.1371/journal.pone.0220688 (PMC6690525; doi:10.1371/journal.pone.0220688)
Supplement: S3 Table — Rest of moderated moderation analyses showed no significant three-way interaction. (DOCX) [file pone.0220688.s004.docx]

S3 Table. Conditional effect of age and gender on the relationship of the perceiving and facilitating MSCEIT branches with expressive suppression, and on the relationship of MSCEIT understanding with cognitive reappraisal. Rest of moderated moderation analyses showed no significant three-way interaction.

|  |  | MSCEIT perceiving Expressive suppression | | MSCEIT facilitating Expressive suppression | | MSCEIT understanding Cognitive reappraisal | |
| --- | --- | --- | --- | --- | --- | --- | --- |
| Gender | Age | Effect | 95% CI  [lower, upper] | Effect | 95% CI  [lower, upper] | Effect | 95% CI  [lower, upper] |
| Men | Lower | -.0213 | [-.0385, -.0040] | -.0249 | [-.0434, -.0064] | -.0007 | [-.0140, .0127] |
| Men | Medium | -.0108 | [-.0214, -.0002] | -.0124 | [-.0237, -.0011] | .0084 | [-.0001, .0168] |
| Men | Higher | -.0003 | [-.0114, .0107] | .0000 | [-.0117, .0117] | .0174 | [.0084, .0264] |
| Women | Lower | -.0007 | [-.0110, .0096] | -.0090 | [-.0193, .0012] | .0085 | [.0000, .0169] |
| Women | Medium | -.0054 | [-.0134, .0027] | -.0142 | [-.0224, -.0060] | .0062 | [-.0009, .0133] |
| Women | Higher | -.0101 | [-.0223, .0022] | -.0193 | [-.0316, -.0070] | .0040 | [-.0070, .0150] |
